# A Few Remarks on Cholera

**Published:** 1866-07

**Authors:** Thos. F. Rochester


BUFFALO

### Medical and Surgical Journal.

VOL. V. JULY, 1866. No. 12.

ART. I—*A few Remarks on Cholera.* BY THOS. F. ROCHESTER, M. D.

Never, perhaps, was the approach of an epidemic disease watched, discussed and feared, more than that of the pestilence which even now has commenced its attack upon the chief city of the United States. This arises from natural and proper anxiety, from ignorance, and from the different and almost opposite views of medical men upon the nature, propagation and treatment of the disease. It is the object of the writer of this paper, to endeavor to disarm the minds of those members of his profession, who have never encountered the disorder, of that doubt and distrust of remedial measures, which is the natural result of the contrariety of opinions, expressed in the pages of the medical journals of the day. The remarks which follow are not based upon any theoretical ideas, nor are they intended to imply any superior knowledge to that possessed by those whose opportunities of treating and observing cholera have extended over two or more of its visitations. They are simply the expression of convictions based upon a large professional experience derived from four epidemics, three in this country and one in Europe. Cholera is probably properly classed as a zymotic affection—emanating from the filthy and densely populated districts of the East Indies, it has at various times and with variable intermissions desolated large portions of the habitable globe—sometimes traveling as it were in a fixed course, from east to west, and sometimes taking irregular flights and pouncing upon distant and isolated localities, scourging them fearfully, and then for a long time entirely subsiding. It has been regarded as an atmospheric, indefinite *something*, capable of extending itself against winds, over mountains, and across large bodies of water. Again it has been thought telluric, an exhalation from the deadly jungles of Siam and Hindostan—and latterly and with more reason, it is looked upon as a disease, however arising, that is extended and propagated by those persons who are exposed to it, and more notably by those who sre attacked by it. In no other manner can it be reasonably explained how it breaks out in mid-ocean. The germ taken from some infected port, breaks out at last, especially under the inviting combination of filth, crowding, bad ventilation and improper food. It is usually characterized by well marked premonitory symptoms; these are a sense of fatigue and lassitude, and a *painless* diarrhœa, the latter having a duration of from a few hours to several days. This diarrhœa, however, does not always pre-exist. The writer has known of at least two instances where violent vomiting was the first intimation of any derangement of health, and in both cases death ensued in less than six hours. During the outbreak of cholera at Suspension Bridge in 1854, we have the statement of Dr. Sanford B. Hunt that the disease was often fatal in a few hours, and where there had been no premonitory diarrhœa whatever. Happily, however, in the vast majority of cases, there is a premonitory diarrhœa. When the disease is fully formed, this is replaced by copious discharges from the stomach or bowels, or from both, of a *serous* fluid, containing more or less white flocculi, and hence called *rice water discharges.* This serous fluid is nearly identical in composition with the serum of the blood, while the flocculi are formed of shreds of mucous membrane and of its epithelia. Great weakness, cramps in the extremities, coldness of the surface, huskiness of the voice, shriveling and moisture and lividity of the skin and entire suppression of urine, with sometimes slow and sometimes frequent, but always feeble pulse, and often entire cessation of the radial pulsation, ensue, and constitute the algid or collapse stage of the disease, and in it death often takes place apparently from syncope. When re-action does occur, it is often succeeded by rapid restoration to health. In some instances, however, and especially in some epidemics, what is termed *the congestive stage* is developed, and this is often fatal. It may be briefly described as a typhus state, with marked cerebral derangement.

The above description, it is believed, presents most of the phenomena of cholera. There are, it is true, a few cases to which it will not fully apply, as collapse with cramps and suppression of urine without either vomiting or purging, but even in these instances, where post mortem examination has been held, the stomach and intestines have been found filled with the characteristic rice water. At this point, the question of the communicability of cholera seems pertinent. Until within the last two years the weight of medical opinion has been opposed to the idea of infection, but the writer feels that it is but just to himself to declare, that for fifteen years he has been one of the few who have held and taught, that cholera, and dysentery also, was probably propagated by the evacuations. The manner in which cholera has been thus far restricted by rigid quarantine regulations, this season, in New York bay, would seem to sustain this view. Not knowing what the morbific poison is, nor its precise derivation, we are also at a loss as to its exact mode of action. One insists that its first effect is upon the nervous centres, another is equally positive that the blood itself is the primary seat of change; another finds the liver and alimentary canal in fault; and still another refers most of the trouble to the respiratory organs and to the circulatory apparatus. The fact is, this terrible poison *acts* upon the entire system, and the attempt to localize its specific place and mode of action has never succeeded, and has probably only made mischief. The information we obtain from necroscopy, relates chiefly to the mucous membrane of the alimentary canal and to the blood. In those who die in the algid stage, the stomach and intestines contain more or less of the *rice water;* the mucous membrane is pale, looking as if it had been macerated, the epithelial surface often being absent, as if it had been dissolved or washed away. The gall bladder usually is well filled with the biliary secretion. The right side of the heart is filled with thick, pasty, dark blood; the left chambers are empty and collapsed. The lungs are increased in specific gravity, made more dense in parts by a kind of atelectasis, and often contain thick, dark, infiltrated blood spots. The red points in the brain are large and deepened in color. The kidneys are dark and congested, and the bladder is empty and contracted. In those who die in the febrile or congestive stage, the mucous membrane of the stomach is covered with a thick, tough secretion, while that of the small and large intestines is red and injected, and in some instances inflammed and even ulcerated in portions of its tract. The plates of Peyer are sometimes found enlarged, and when this is the case, the corresponding mesenteric glands are red, softened, and also enlarged. The blood in the right side of the heart is less thick and pasty than in the algid stage, and the left chambers are not found entirely empty. The kidneys are red, but not engorged, and a small amount of urine is generally found in the bladder. The membranes of the brain are injected, and there is usually a little fluid in the ventricles. The lungs present evidences of passive engorgement.—From the phenomena of the disease and from the post mortem appearances, is it not evident that there is intense *gastro-enteritis*, painless because attended with copious secretion, and, will not this "serous diarrhœa" explain the condition of the blood and of the circulatory and respiratory apparatus, the nervous prostration and the suspension of the urinary secretion? Of course there is a cause, an unknown cause, that induces this tremendous gastro-enteric secretion, and this morbific force is sometimes so intense and overwhelming, that it induces death from *shock*, as in Muscat, where, "from the first apparent seizure, ten minutes only elapsed before life was extinct." But although there are doubtless many exceptional cases, it certainly seems to the writer, that in the large majority of instances the ratio of mortality corresponds with the amount and duration of the "serous diarrhœa," Dr. Johnson of London, to the contrary notwithstanding. *If it is* true that by vomiting and purging the choleraic poison is eliminated, why do all sensible men urge attention to the premonitory diarrhœa? Why is an attack of cholera induced by improper diet? and why is an active and irritating cathartic distinctly the exciting cause of many fatal cases?

(TO BE CONCLUDED IN AUGUST NUMBER.)

MURRAIN IN PALESTINE.—From a report received from Captain Wilson, in Damascus, it appears that the murrain among the cattle still rages violently in the north of Palestine.

